# A commentary on ‘Objective measurement of retention of laparoscopic skills: a prospective cohort study’

**DOI:** 10.1097/JS9.0000000000000493

**Published:** 2023-05-24

**Authors:** Si-Un Frank Chiu, Hui-Ming Lee, Chong-Chi Chiu

**Affiliations:** a Department of Computer Science; b Department of Economics, Brown University, Providence, Rhode Island, USA; c Department of General Surgery; d Department of Medical Education and Research, E-Da Cancer Hospital; e School of Medicine, College of Medicine, I-Shou University, Kaohsiung, Taiwan

*Dear Editor,*

We read with interest the prospective cohort study by Rahimi *et al*.^1^. They aimed to investigate the retention of laparoscopic technical skills in surgical residents 4 months after completing a Basic Laparoscopy Course. The study concluded that skill deterioration was present for the Post and Sleeve and the ZigZag loop tasks regarding force, motion, and time parameters. Finally, the authors recommended incorporating maintenance training to preserve acquired laparoscopic skills. Although the study’s findings are significant, some demerits in the design compromise its validity and applicability.

Firstly, a control group is necessary to establish a causal relationship between completing the Basic Laparoscopy Course and declining technical skills. However, this study lacked a control group, which made it difficult to determine if the observed deterioration was due to the passage of time or if it was a result of insufficient maintenance training. Moreover, it was difficult to rule out other confounding variables that could have influenced the results, such as the learning environment, the trainers’ quality, and the tasks’ complexity.

Secondly, the study was based on a relatively small sample size of 29 participants from 12 Dutch training hospitals, limiting the results’ generalizability. The study’s small sample size also reduced the statistical power of the analysis and increased the likelihood of type II errors, which meant that the study might have failed to detect fundamental differences between the groups due to a lack of statistical power. Furthermore, this study did not consider the individual differences among the residents. Some residents may have already had experience with laparoscopic surgery or have different learning abilities, which could have influenced their skill retention differently. Additionally, the study only used objective measurements to evaluate laparoscopic skills. While objective measures are essential^2,3^, subjective evaluations by experienced surgeons can also be valuable in assessing surgical skills^4^. Therefore, the study’s conclusions need to be more generalized and reflect the reality of surgical practice.

Thirdly, the study only measured the retention of technical skills using force, motion, and time parameters. While these are essential for assessing technical skills, critical laparoscopic surgery’s cognitive and nontechnical aspects, such as situational awareness, decision-making, communication, teamwork, and leadership^5^, must be captured. These aspects are critical for patient safety but should be addressed in this laparoscopic training and judgment program. Therefore, a more comprehensive assessment of laparoscopic technical skills, cognitive and nontechnical domains, is needed to provide a complete picture of the trainee’s competence.

Fourthly, the study’s duration was only 4 months, a relatively short period to assess skill retention. Laparoscopic technical skills may deteriorate over a more extended period, and a longer follow-up would provide more insight into the durability of acquired skills. Future studies should consider more extended follow-up periods to determine the optimal frequency and duration of maintenance training required to preserve laparoscopic technical skills.

Although the authors recommended the incorporation of maintenance training to preserve acquired laparoscopic skills, they neither evaluated the impact of maintenance training on skill retention nor provided any evidence to support this claim. Thus, future studies should investigate the effectiveness of different maintenance training strategies, such as simulator training, coaching, and feedback, in preserving laparoscopic technical skills. Besides, this study should explore why surgical residents failed to maintain their technical skills. Understanding the underlying causes of skill deterioration makes designing effective maintenance training programs easier in the future. Finally, the authors should investigate the factors contributing to skill decay and identify effective strategies to counteract them.

In conclusion, this study provides valuable insights into retaining laparoscopic technical skills in surgical residents. However, its investigation faces some limitations that compromise its validity and generalizability. Future studies should overcome these limitations and provide a more comprehensive understanding of laparoscopic technical skills retention and the role of maintenance training in preserving them. Only then can we ensure surgical trainees acquire and maintain the necessary skills to provide their patients with safe and effective laparoscopic surgery.

## Ethical approval

This is only a commentary, not research involving patients. No ethical approval is required.

## Consent

This is only a commentary, not research involving patients. No patient consent is required.

## Sources of funding

This is a commentary. We have no funding for our commentary.

## Author contribution

S.-U.F.C.: conceptualization and writing the original draft; H.-M.L.: validation; C.-C.C.: conceptualization, supervision, submission, and correspondence.

## Conflicts of interest disclosure

There are no conflicts of interest in our commentary.

## Research registration unique identifying number (UIN)

This is a commentary on a study published in ‘*International Journal of Surgery*’. No UIN is required.

## Guarantor

Professor Chong-Chi Chiu.

## Data availability statement

This is only a commentary on a study published in ‘*International Journal of Surgery*’. There is no research data in our commentary.

## Provenance and peer review

This paper was not invited.
